# Undifferentiated Hepatic Sarcoma: A Rare Case

**DOI:** 10.7759/cureus.25895

**Published:** 2022-06-13

**Authors:** James R Pellegrini, Jose R Russe-Russe, Tate W Higgins, Shino Prasandhan, Nausheer Khan

**Affiliations:** 1 Internal Medicine, Nassau University Medical Center, East Meadow, USA; 2 Gastroenterology, Nassau University Medical Center, East Meadow, USA

**Keywords:** immunohistochemical, gastrointestinal neoplasms, gastrointestinal, primary hepatic sarcoma, oncological imaging, clinical case report, onco, onco-surgery, undifferentiated sarcoma, hepatic sarcoma

## Abstract

Primary hepatic undifferentiated pleomorphic sarcoma (UPS) is a rare malignant mesenchymal tumor with a nonspecific clinical and radiologic presentation. Primary hepatic UPS is often a diagnosis of exclusion made by multiple immunohistological testing that rules out hepatic, hematologic, neural, and epithelial origin. Stains for mesenchymal origin are usually the only positive stain and do not demonstrate evidence of specific mesenchymal cell differentiation. We report a case of a 56-year-old male with no significant past medical history that presented with complaint of epigastric abdominal pain of six months duration. A computed tomography (CT) scan of the abdomen and pelvis exhibited numerous hepatic masses involving right and left hepatic lobe. A CT-guided core needle biopsy discovered undifferentiated/pleomorphic sarcoma. Histomorphology showed spindle cell neoplasm without recognizable hepatic tissue. Immunohistochemistry (IHC) stains were positive for smooth muscle actin (SMA) but failed to establish a more specific histogenesis. Furthermore, IHC stains revealed spindle neoplastic cells with focal and patchy positive h-caldesmon (approximately 10-15% of neoplastic cells), and negative for desmin. Given these results, the diagnosis of undifferentiated/pleomorphic sarcoma was established. It is imperative to consider UPS in the differential diagnosis of large liver lesions without evidence of differentiation. Early identification of this rare tumor can prevent the possibility of distant metastasis and improve survival among patients.

## Introduction

Primary hepatic undifferentiated pleomorphic sarcoma (UPS) is a rare malignant mesenchymal tumor with a nonspecific clinical presentation and radiologic imaging. Patients present with a history of weight loss, fever, jaundice, malaise, right upper quadrant pain, and palpable abdominal mass [1]. Abdominal imaging studies such as computerized tomography (CT) and ultrasound (U/S) are only able to reveal a mass and are nonspecific for UPS. As a result, primary hepatic UPS is a diagnosis of exclusion made by immunohistological testing that rules out hepatic, gastrointestinal stromal tumor (GIST), hematological, neural, and epithelial origin [2]. Stains for mesenchymal origin are usually the only positive stain and do not indicate mesenchymal cell differentiation [2]. Here we present a case of a patient that was found to have this rare malignant mesenchymal tumor and presented with nonspecific signs and symptoms.

This article was previously presented as an abstract to the American College of Gastroenterology (ACG) Annual Scientific Meeting in Las Vegas, Nevada, in October 2021.

## Case presentation

A 56-year-old male with no significant past medical history presented to the gastroenterology clinic with complaint of nonradiating epigastric pain of six months duration. The patient described the pain as nonradiating, burning in nature, and worse postprandial. The patient also reported a 30-pound unintentional weight loss in the past six months and straining on defecation. The patient denied nausea, vomiting, diarrhea, recent travel, dysphagia, melena, and hematochezia. Physical examination revealed a thin middle-aged male with a distended abdomen, hepatomegaly, mild tenderness to palpation of the right upper quadrant with no guarding, rigidly or rebound tenderness. Initial laboratory analysis showed decreased hemoglobin, elevated aspartate aminotransferase, elevated alanine aminotransferase, and elevated alkaline phosphatase (Table 1).

**Table 1:** Laboratory results of the patient.

Test	Unit	Result	Reference
Hemoglobin	g/dL	12.2	13-17
Aspartate aminotransferase	u/L	116	5-40
Alanine aminotransferase	u/L	62	7-55
Alkaline phosphatase	u/L	512	44-147

**Figure 1:**
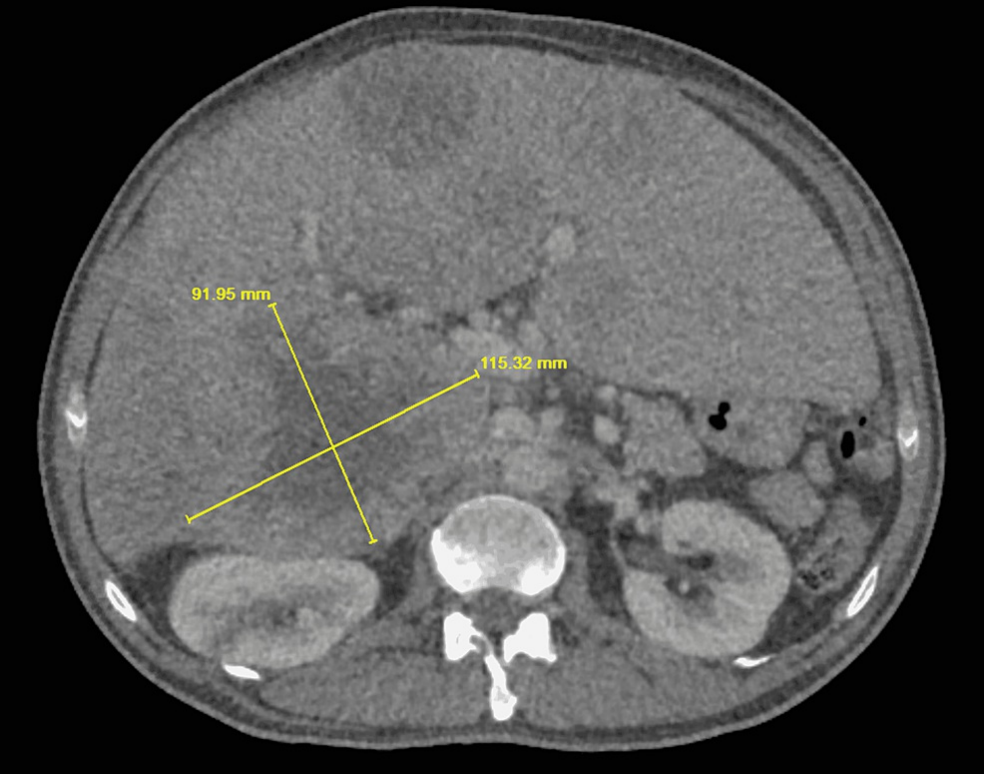
Right hepatic lobe is measuring 11.5 x 9.2 cm with bulging of the liver capsule and compressing the inferior vena cava.

A CT scan of the abdomen and pelvis exhibited innumerable bilateral nodular densities of variable size. Numerous hepatic masses involving right and left hepatic lobe, with several lesions distorting the capsule and displacing the right kidney posteriorly were seen. One of the large lesions in the right hepatic lobe was measured to be 11.5 x 9.2 cm with bulging of the liver capsule and compression of the inferior vena cava (Figure 1). Other notable findings on CT include a 3.5 x 2.0 cm soft lesion compressing the right kidney posterior parenchyma, a 1.6 x 1.4 cm left kidney midpole cortical solid lesion, thickening of the left adrenal gland, an expansive osteolytic lesion in the right transverse process of the L2 vertebral body, and a 1.1 cm lesion at posterior aspect of L5 vertebral body protruding into the spinal canal.

**Figure 2:**
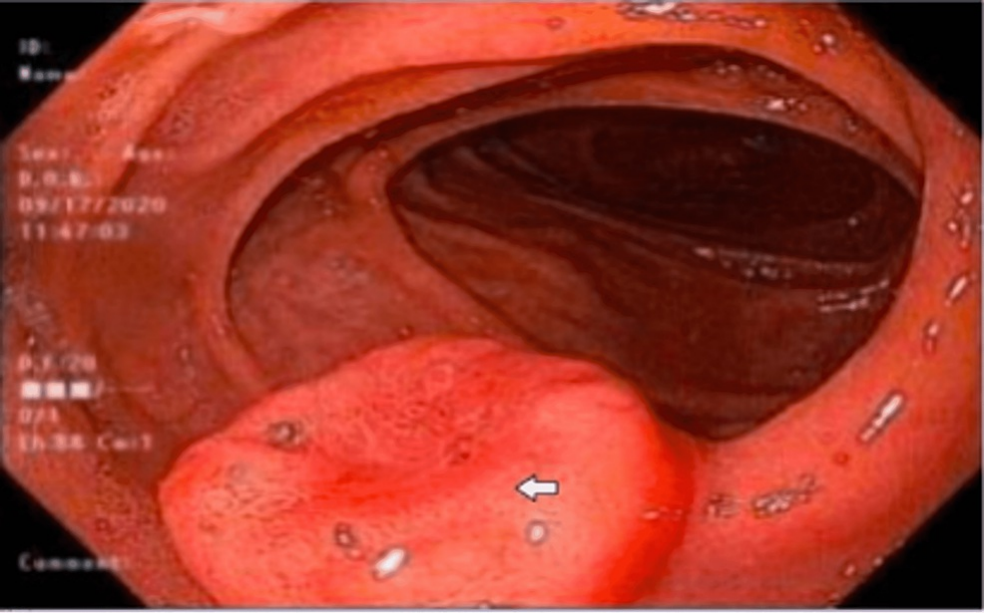
Duodenal nodule as seen on EGD. EGD: esophagogastroduodenoscopy

**Figure 3:**
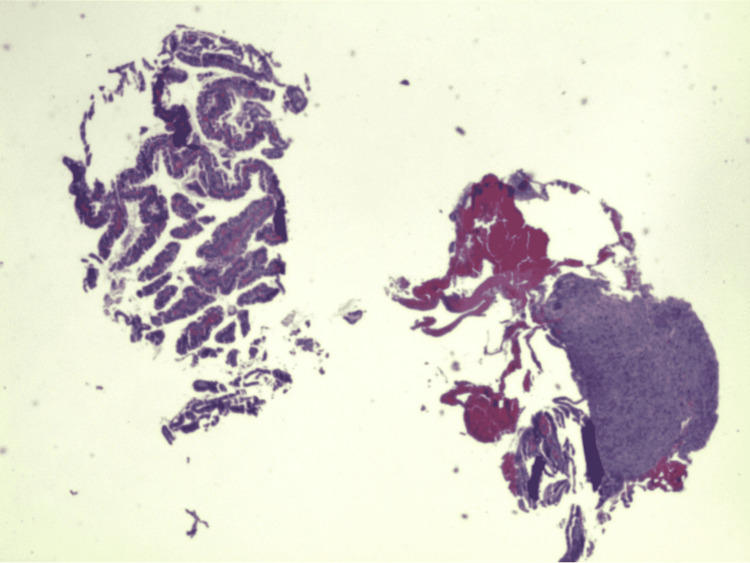
Biopsy of duodenal nodules revealing spindle cell neoplasm.

**Figure 4:**
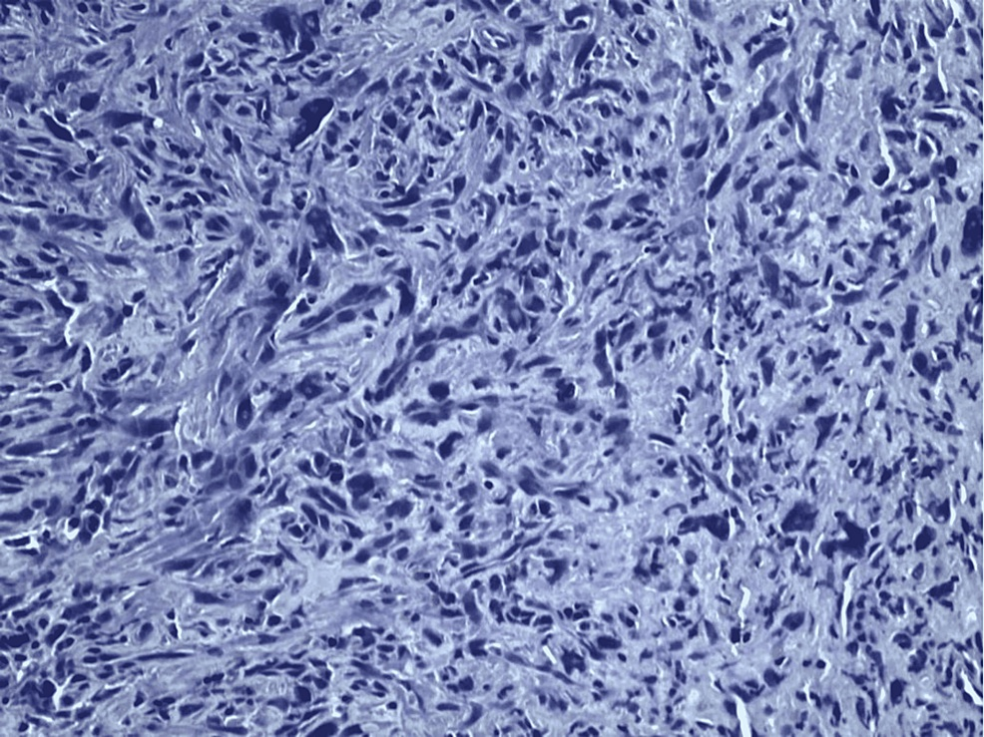
Biopsy of duodenal nodules showing moderate eosinophilia.

**Figure 5:**
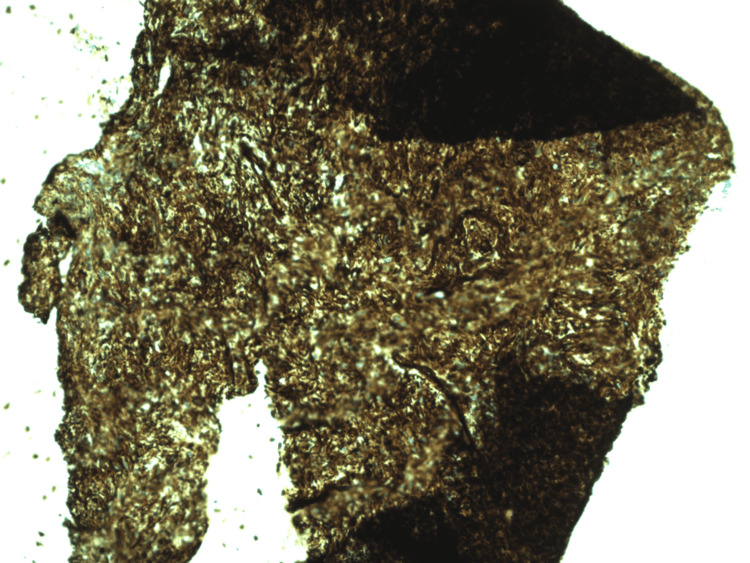
Immunohistochemistry (IHC) of the duodenal nodules showed uncharacterized vimentin positive spindle cell neoplasm.

**Figure 6:**
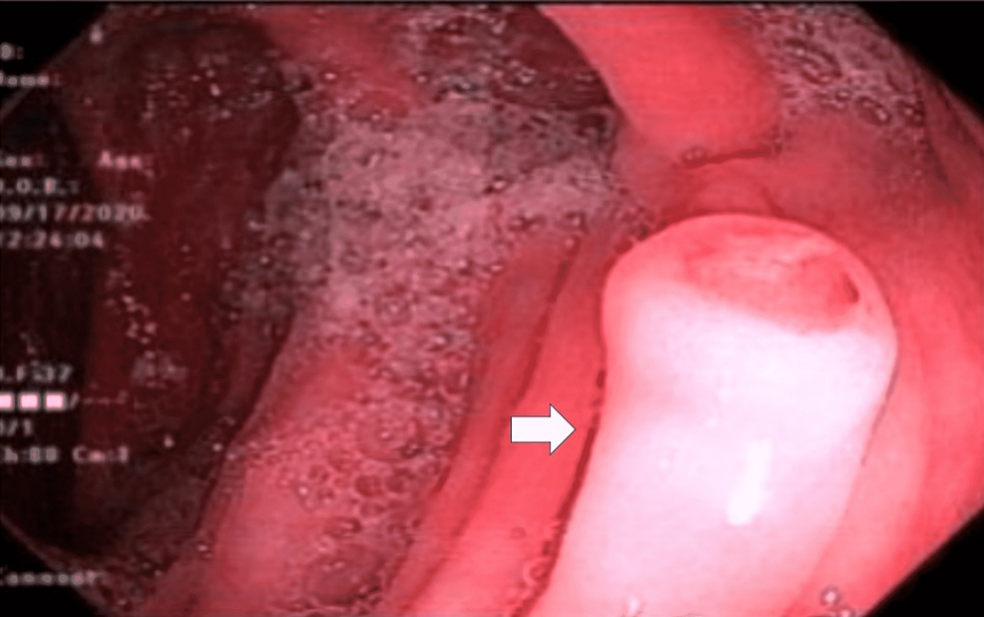
Colonoscopy showing 1 cm nodule in the ascending colon.

**Figure 7:**
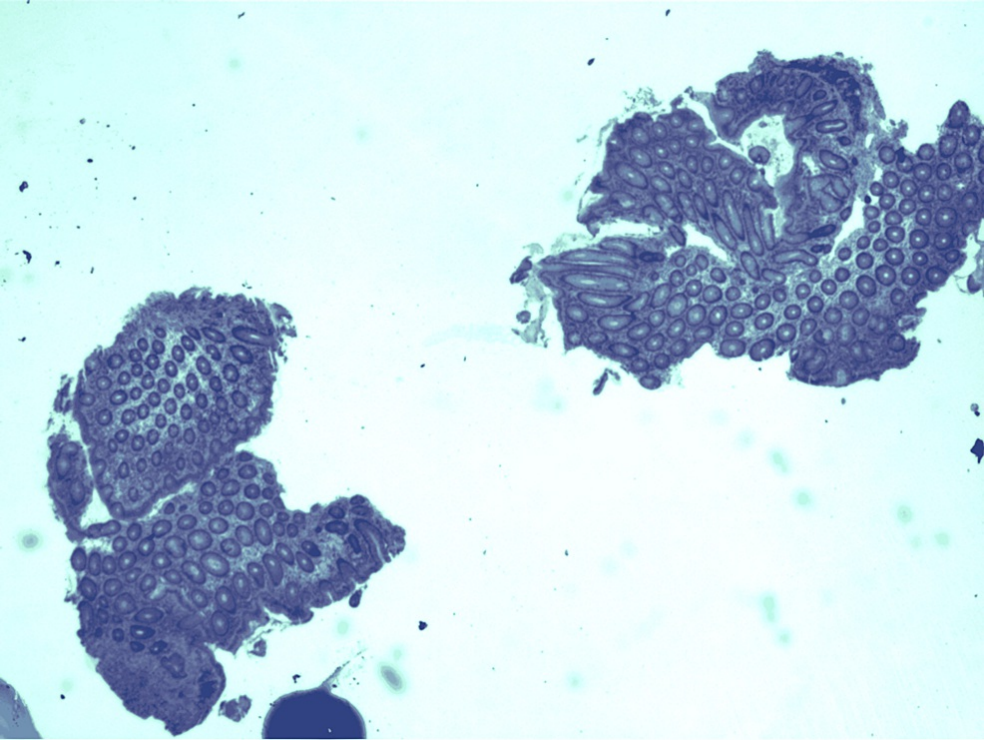
Biopsy of the ascending colon nodule showing foci of spindle cell nodular lesions consistent with spindle cell neoplasm.

**Figure 8:**
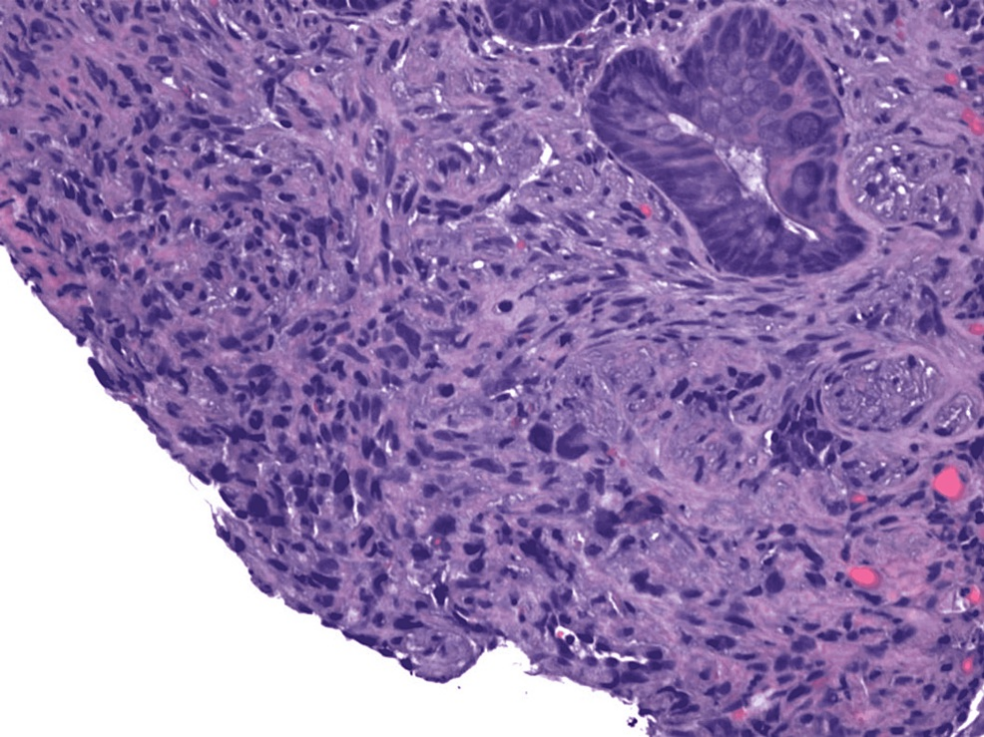
Biopsy of the ascending colon nodule with hyperplastic changes.

An esophagogastroduodenoscopy (EGD) was performed and showed a subcentimeter nodule in the duodenal bulb, a 1 cm nodule in the second portion of the duodenum, and gastritis (Figure 2). Biopsy of the duodenal nodules was found to have spindle cell neoplasm with moderate eosinophilia (Figures 3, 4). IHC of the duodenal nodules showed uncharacterized vimentin positive spindle cell neoplasm (Figure 5). A colonoscopy was performed that revealed a 1 cm nodule in the ascending colon and internal hemorrhoids (Figure 6). Biopsy of the ascending colon nodule showed foci of spindle cell nodular lesions consistent with spindle cell neoplasm with hyperplastic changes (Figures 7, 8).

**Figure 9:**
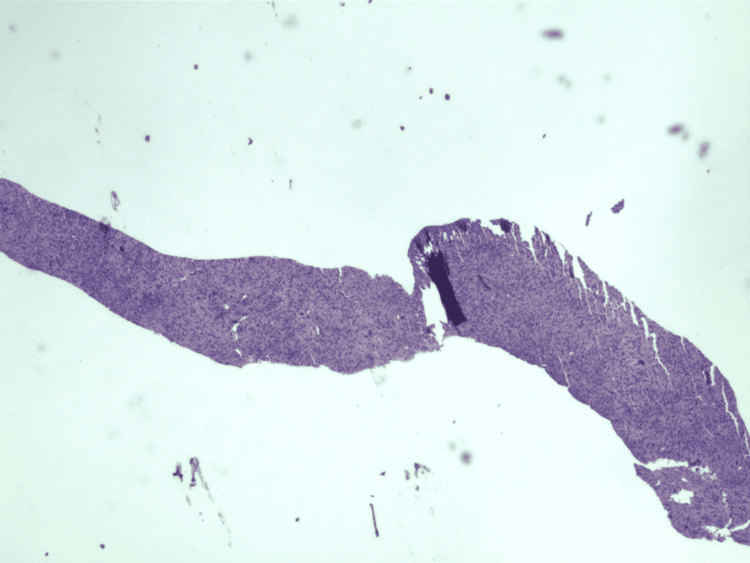
Liver biopsy discovering sarcoma, undifferentiated/pleomorphic SMA positive. SMA: smooth muscle actin

**Figure 10:**
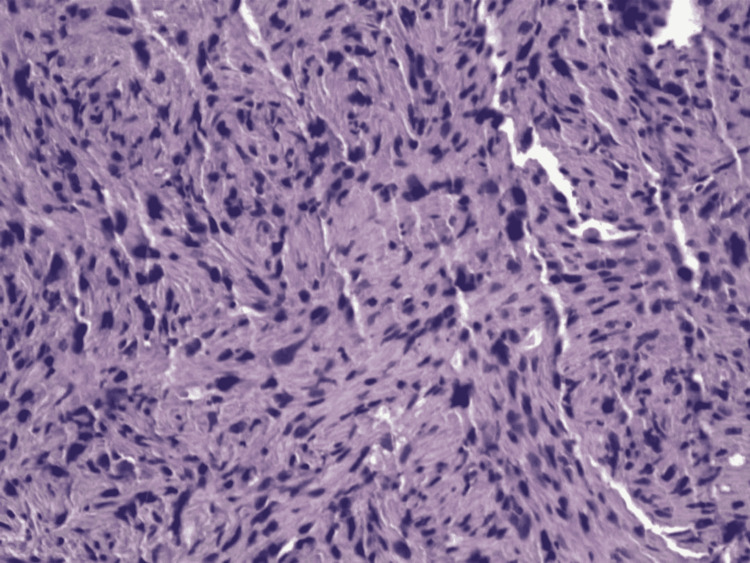
Histomorphology revealing spindle cell neoplasm without recognizable hepatic tissue.

A biopsy sample of one of the hepatic masses was obtained via CT-guided core needle biopsy. Immunohistochemistry (IHC) was smooth muscle actin (SMA) positive (Figure 9). Histomorphology showed spindle cell neoplasm without recognizable hepatic tissue (Figure 10). A wide battery of stains failed to establish a more specific histogenesis. We found spindle neoplastic cells that were focal and patchy positive for h-caldesmon (approximately 10-15% of neoplastic cells), and negative for desmin. Given these results, the diagnosis of undifferentiated/pleomorphic sarcoma was established.

Further imaging with magnetic resonance imaging (MRI) disclosed metastasis to the spinal cord and brain. The patient was medically optimized and advised to follow-up in the outpatient gastroenterology and oncology clinics. The latest oncology note states the patient declined chemotherapy. Patient consent for publication was received.

## Discussion

Sarcomas are malignant cells of mesenchymal origin characterized by a heterogeneous pattern on histologic examination. Soft tissue sarcomas are extremely rare, accounting for less than 1% of all cancers [2]. Previously, UPS was known as malignant fibrous histiocytoma (MFH). UPS is typically located in the extremities; however, has been found in visceral organs as well [3]. To date, there are fewer than 200 cases reported of primary hepatic UPS in the medical literature [2]. This type of malignancy is extremely rare and accounts for about 0.1-2% of all forms of hepatic cancers [2].

UPS was found to be rich in histiocytes with a storiform growth pattern, which was the basis for its first name MFH. The current definition of UPS requires that it does not demonstrate evidence of specific mesenchymal cell differentiation. There are four main types of histological patterns seen in UPS; myxoid, inflammatory, giant cell, and storiform pleomorphic [4].

Primary hepatic UPS typically presents in late adulthood, at a mean age of 58 years [1]. There has been no predominant sex distribution described in medical literature [1]. Most reported cases of primary hepatic UPS have been associated with radiation exposure; however, a large majority of cases have no known etiologic link. Symptoms of primary hepatic UPS are generally nonspecific. Patients frequently complain of abdominal pain, weight loss, anorexia, fever, jaundice, malaise, and a palpable abdominal mass [4,5]. Diagnostic tools including radiological imaging and laboratory studies are insufficient to make the diagnosis of UPS. Laboratory results are typically unremarkable until the tumor begins to compress nearby structures and often lack usefulness for a preoperative diagnosis. Abdominal imaging studies such as CT and U/S are able to reveal a mass, with the mean diameter in reported cases of primary hepatic UPS being about 12.2 cm [4].

As a result of these diagnostic challenges, primary hepatic UPS is ordinarily a diagnosis of exclusion and made by multiple immuno-histological tests that rule out hepatic, gastrointestinal stromal tumor (GIST), hematological, neural, and epithelial origin [6]. Stains for mesenchymal origin are usually the only positive stain and do not demonstrate evidence of specific mesenchymal cell differentiation [6,7]. The presence of tissue eosinophilia on IHC can be indicative of metastatic disease, as the result of a primary disease or as a paraneoplastic syndrome [8].

The standard treatment for primary hepatic UPS is radical tumor resection with clean margins, with the goal of complete eradication of the disease. The effects of chemotherapy and radiotherapy on UPS tumors are unclear [7]. Doxorubicin, an anthracycline antibiotic with antineoplastic activity, has shown promise in improving prognosis and radiation therapy may prevent local tumor recurrence [9]. There are multiple surgical procedures depending on the location of the tumor, and a standard procedure has not been established. 

Due to the aggressive nature of UPS and its extraordinary potential for local recurrence and distant metastasis, the prognosis for patients is poor [7,9]. Some cases have reported favorable outcomes if the diagnosis is made early and there is no evidence of metastasis [9].

## Conclusions

Primary hepatic UPS is a rare malignant mesenchymal tumor with a nonspecific clinical and radiologic presentation. It is imperative to consider UPS in the differential diagnosis of large liver lesions without evidence of differentiation. Early identification of this rare tumor can prevent the possibility of distant metastasis and improve survival.
